# Supplementary material for: The proteomic landscape and temporal dynamics of human and mouse gastruloid development
Source: Nat Cell Biol. 2026 Apr 24;28(5):1015–30. doi: 10.1038/s41556-026-01937-5 (PMC13179132; doi:10.1038/s41556-026-01937-5)
Supplement: Supplementary file 1 — Reporting Summary [file 41556_2026_1937_MOESM1_ESM.pdf]

nature portfolio

Corresponding author(s): Jay Shendure
Lea Starita
Nobuhiko Hamazaki
Devin Schweppe
Riddhiman K. Garge

Last updated by author(s): Jan 7, 2026

Reporting Summary

Nature Portfolio wishes to improve the reproducibility of the work that we publish. This form provides structure for consistency and transparency in reporting. For further information on Nature Portfolio policies, see our Editorial Policies and the Editorial Policy Checklist.

Statistics

For all statistical analyses, confirm that the following items are present in the figure legend, table legend, main text, or Methods section.

n/a	Confirmed	
☐	☒	The exact sample size (*n*) for each experimental group/condition, given as a discrete number and unit of measurement
☐	☒	A statement on whether measurements were taken from distinct samples or whether the same sample was measured repeatedly
☐	☒	The statistical test(s) used AND whether they are one- or two-sided<br>*Only common tests should be described solely by name; describe more complex techniques in the Methods section.*
☒	☐	A description of all covariates tested
☐	☒	A description of any assumptions or corrections, such as tests of normality and adjustment for multiple comparisons
☐	☒	A full description of the statistical parameters including central tendency (e.g. means) or other basic estimates (e.g. regression coefficient) AND variation (e.g. standard deviation) or associated estimates of uncertainty (e.g. confidence intervals)
☐	☒	For null hypothesis testing, the test statistic (e.g. *F*, *t*, *r*) with confidence intervals, effect sizes, degrees of freedom and *P* value noted<br>*Give P values as exact values whenever suitable.*
☒	☐	For Bayesian analysis, information on the choice of priors and Markov chain Monte Carlo settings
☒	☐	For hierarchical and complex designs, identification of the appropriate level for tests and full reporting of outcomes
☐	☒	Estimates of effect sizes (e.g. Cohen's *d*, Pearson's *r*), indicating how they were calculated

Our web collection on statistics for biologists contains articles on many of the points above.

Software and code

Policy information about availability of computer code

Data collection	Basecall files were converted to fastq formats using bcl2fastq (Illumina) and demultiplexed on the i5 and i7 indexes. Adapter trimming was performed using Trimmomatic v0.39. Depending on the species, trimmed reads were then aligned using STAR to either the human GRCh38 or mouse GRCm39 reference assemblies. Count matrices were then generated with bam files using FeatureCounts.For proteomics data, raw files were searched against the relevant annotated proteome from Uniprot. Comet search algorithm was utilized to match peptides to spectra. Peptide-spectrum matches (PSMs) were filtered to a 1% false discovery rate (FDR). Proteins were filtered to an FDR of 1%. For quantitation, PSMs were required to have a summed TMT reporter ion signal-to-noise ≥100.For further detail, please see the Methods section.
Data analysis	The code used to analyze the data, and generate figures are deposited in the following GitHub repository- https://github.com/bbi-lab/Temporal-Gastrulomics<br>The following freely available R packages were used for data analysis:<br>Tidyverse- https://cran.r-project.org/web/packages/tidyverse/index.html<br>Scales- https://cran.r-project.org/web/packages/scales/index.html<br>ggrepel- https://cran.r-project.org/web/packages/ggrepel/index.html<br>ggrastr- https://cran.r-project.org/web/packages/ggrastr/index.html<br>cowplot- https://cran.r-project.org/web/packages/cowplot/index.html<br>RColorBrewer- https://cran.r-project.org/web/packages/RColorBrewer/index.html<br>ggplot2- https://cran.r-project.org/web/packages/ggplot2/index.html<br>ggridges- https://cran.r-project.org/web/packages/ggridges/index.html<br>ggpmisc- https://cran.r-project.org/web/packages/ggpmisc/index.html<br>GGally- https://cran.r-project.org/web/packages/GGally/index.html

psych- https://cran.r-project.org/web/packages/psych/index.html
ggpubr- https://cran.r-project.org/web/packages/ggpubr/index.html
reshape2- https://cran.r-project.org/web/packages/reshape2/index.html
umap- https://cran.r-project.org/web/packages/umap/index.html
Seurat- https://cran.r-project.org/web/packages/Seurat/index.html
EnvStats- https://cran.r-project.org/web/packages/EnvStats/index.html
pheatmap- https://cran.r-project.org/web/packages/pheatmap/index.html
dendsort- https://cran.r-project.org/web/packages/dendsort/index.html
amap- https://cran.r-project.org/web/packages/amap/index.html
Matrix- https://cran.r-project.org/web/packages/Matrix/index.html
igraph- https://cran.r-project.org/web/packages/igraph/index.html
DeSeq2- https://bioconductor.org/packages/release/bioc/html/DESeq2.html
conflicted- https://cran.r-project.org/web/packages/conflicted/index.html

For manuscripts utilizing custom algorithms or software that are central to the research but not yet described in published literature, software must be made available to editors and reviewers. We strongly encourage code deposition in a community repository (e.g. GitHub). See the Nature Portfolio guidelines for submitting code & software for further information.

Data

Policy information about availability of data

All manuscripts must include a data availability statement. This statement should provide the following information, where applicable:

- Accession codes, unique identifiers, or web links for publicly available datasets
- A description of any restrictions on data availability
- For clinical datasets or third party data, please ensure that the statement adheres to our policy

DATA AVAILABILITY
RNA-seq data have been deposited to the Gene Expression Omnibus (GEO) database with the identifier GSE273813. Mass spectrometry proteomics data have been deposited to the ProteomeXchange Consortium via the PRIDE partner repository with the dataset identifier PXD054460. Reviewers can access these data through PRIDE using the account details: Username: reviewer_pxd054460@ebi.ac.uk; Password: 4lQJ5v6pqvGs.

CODE AVAILABILITY
All supporting scripts and code have been deposited onto the following repository at https://github.com/bbi-lab/Temporal-Gastrulomics. All processed data are available through the web application at: https://gastruloid.brotmanbaty.org/.

No experimental data were excluded from the analyses. Sequencing and spectrometry data exclusion criteria is outlined in the Methods, including filtering out the substandard reads and spectra, following general practices in genomics and proteomics.

Research involving human participants, their data, or biological material

Policy information about studies with human participants or human data. See also policy information about sex, gender (identity/presentation), and sexual orientation and race, ethnicity and racism.

Reporting on sex and gender	N/A
Reporting on race, ethnicity, or other socially relevant groupings	N/A
Population characteristics	N/A
Recruitment	N/A
Ethics oversight	N/A

Note that full information on the approval of the study protocol must also be provided in the manuscript.

Field-specific reporting

Please select the one below that is the best fit for your research. If you are not sure, read the appropriate sections before making your selection.

☒ Life sciences ☐ Behavioural & social sciences ☐ Ecological, evolutionary & environmental sciences

For a reference copy of the document with all sections, see nature.com/documents/nr-reporting-summary-flat.pdf

Life sciences study design

All studies must disclose on these points even when the disclosure is negative.

Sample size	No statistical methods were used to pre-determine sample sizes but the sample sizes used in this study are comparable to those reported in previous publications (please see main text for references)
Data exclusions	Data exclusion criteria for transcriptomic and proteomic assays and datasets are outlined in the methods section, including the filtering metrics, all of which are in line with general practices in the field.
Replication	All gastruloid samples were collected within the same experimental batch to avoid confounding issues from batch effects. Samples profiled using proteomics were cultured in triplicate while samples profiled using transcriptomics were cultured in duplicate. All other experiments were repeated in triplicate.
Randomization	Human RA-gastruloids and mouse conventional gastruloids were randomly selected within each timepoint before multi-omics phenotyping.
Blinding	The investigators were not blinded to allocation during experiment and outcome assessment.

Reporting for specific materials, systems and methods

We require information from authors about some types of materials, experimental systems and methods used in many studies. Here, indicate whether each material, system or method listed is relevant to your study. If you are not sure if a list item applies to your research, read the appropriate section before selecting a response.

Materials & experimental systems

n/a	Involved in the study	
☐	☒	Antibodies
☐	☒	Eukaryotic cell lines
☒	☐	Palaeontology and archaeology
☒	☐	Animals and other organisms
☒	☐	Clinical data
☒	☐	Dual use research of concern
☒	☐	Plants

Methods

n/a	Involved in the study	
☒	☐	ChIP-seq
☒	☐	Flow cytometry
☒	☐	MRI-based neuroimaging

Antibodies

Antibodies used	Phospho-Histone H2A.X (Ser139) (20E3) Rabbit Monoclonal Antibody 5763. Dilution- 1:50<br>Anti-ATP5A antibody ab14748. Dilution- 1:1000<br>Anti-SOX2 Antibody AB5603. Dilution- 1:100<br>Donkey anti-Mouse IgG (H+L) Highly Cross-Adsorbed Secondary Antibody, Alexa Fluor™ 647 A-31571. Dilution- 1:500<br>Donkey anti-Rabbit IgG (H+L) Highly Cross-Adsorbed Secondary Antibody, Alexa Fluor™ Plus 488 A32790. Dilution- 1:500<br>Donkey anti-Rabbit IgG (H+L) Highly Cross-Adsorbed Secondary Antibody, Alexa Fluor™ Plus 555 A32794. Dilution- 1:500
Validation	Donkey anti-Mouse IgG (H+L) Highly Cross-Adsorbed Secondary Antibody, Alexa Fluor™ 647 A-31571 Validated in HeLa cells by manufacturer https://www.thermofisher.com/antibody/product/Donkey-anti-Mouse-IgG-H-L-Highly-Cross-Adsorbed-Secondary-Antibody-Polyclonal/A-31571<br>Donkey anti-Rabbit IgG (H+L) Highly Cross-Adsorbed Secondary Antibody, Alexa Fluor™ Plus 488 A32790 Validated in HEK293 cells by manufacturer https://www.thermofisher.com/antibody/product/A32790.html<br>Donkey anti-Rabbit IgG (H+L) Highly Cross-Adsorbed Secondary Antibody, Alexa Fluor™ Plus 555 A32794 Validated in A549 cells by manufacturer https://www.thermofisher.com/antibody/product/Donkey-anti-Rabbit-IgG-H-L-Highly-Cross-Adsorbed-Secondary-Antibody-Polyclonal/A32794

Eukaryotic cell lines

Policy information about [cell lines and Sex and Gender in Research]

Cell line source(s)	The RUES2-GLR line was provided by Dr. Ali H. Brivanlou (Rockefeller University). Naive and primed H9 ESCs were kindly provided by Dr. Austin Smith (University of Exeter). Mouse ESC line E14Tg2a was obtained from Dr. Christian Schroeter (Max Planck Institute).
Authentication	Activities of three markers SOX2, TBXT, and SOX17 (tagged with mCitrine, mCerulean, and tdTomato respectively) were monitored using fluorescence microscopy.
Mycoplasma contamination	Cell lines used in this study were not tested for Mycoplasma contamination

Commonly misidentified lines (See ICLAC register)	No commonly misidentified cell lines were used.

Plants

Seed stocks	N/A
Novel plant genotypes	N/A
Authentication	N/A
